# Transthyretin interacts with actin regulators in a *Drosophila* model of familial amyloid polyneuropathy

**DOI:** 10.1038/s41598-020-70377-4

**Published:** 2020-08-12

**Authors:** Marina I. Oliveira da Silva, Carla S. Lopes, Márcia A. Liz

**Affiliations:** 1 Instituto de Ciências Biomédicas Abel Salazar (ICBAS), Universidade do Porto, Porto, Portugal; 2 Neurodegeneration Group, Instituto de Biologia Molecular e Celular (IBMC) and Nerve Regeneration Group, Instituto de Investigação e Inovação em Saúde (i3S), Universidade do Porto, Porto, Portugal; 3 Neurodevelopment and Degeneration Group, Instituto de Biologia Molecular e Celular (IBMC) and Glial Cell Biology, Instituto de Investigação e Inovação em Saúde (i3S), Universidade do Porto, Porto, Portugal

**Keywords:** Diseases of the nervous system, Molecular neuroscience

## Abstract

Familial amyloid polyneuropathy (FAP) is a neurodegenerative disorder whose major hallmark is the deposition of mutated transthyretin (TTR) in the form of amyloid fibrils in the peripheral nervous system (PNS). The exposure of PNS axons to extracellular TTR deposits leads to an axonopathy that culminates in neuronal death. However, the molecular mechanisms underlying TTR-induced neurodegeneration are still unclear, despite the extensive studies in vertebrate models. In this work we used a *Drosophila* FAP model, based on the expression of the amyloidogenic TTR (V30M) in the fly retina, to uncover genetic interactions with cytoskeleton regulators. We show that TTR interacts with actin regulators and induces cytoskeleton alterations, leading to axonal defects. Moreover, our study pinpoints an interaction between TTRV30M and members of Rho GTPase signaling pathways, the major actin regulators. Based on these findings we propose that actin cytoskeleton alterations may mediate the axonopathy observed in FAP patients, and highlight a molecular pathway, mediated by Rho GTPases, underlying TTR-induced neurodegeneration. We expect this work to prompt novel studies and approaches towards FAP therapy.

## Introduction

Familial amyloid polyneuropathy (FAP) is a fatal autosomal dominant disease characterized by the extracellular deposition of amyloid fibrils of mutated transthyretin (TTR), particularly in the peripheral nervous system (PNS)^1,2^. As a consequence of TTR deposition, a dying-back axonopathy develops which is followed by neuronal degeneration in advanced disease stages^3^. Physiologically, TTR is mainly synthesized in the liver and choroid plexus of the brain, and secreted to the blood and cerebrospinal fluid (CSF)^4^. TTR expression is also found in the retinal pigment of the eye and in pancreas^5,6^. TTR most acknowledged functions are the transport of thyroxine and retinol^7^. However, it is also an important player in nerve biology being able to increase axon regeneration after sciatic nerve crush and to promote neurite outgrowth of PNS neurons^8^*.*

More than one hundred TTR variants have been reported, being TTRV30M the most frequent mutation associated to FAP. TTRV30M patients develop a progressive peripheral neuropathy usually accompanied by autonomic dysfunction, although TTR amyloid deposits are also found in the eye, kidney, heart and gastrointestinal tract^3^. Central nervous system (CNS) deposits, although rare, have also been reported^9^.

How TTR extracellular aggregates induce intracellular events in FAP is not yet fully understood. Although the dying-back axonal degeneration might suggest a disturbance of the distal cytoskeleton, as a consequence of TTR deposition, cytoskeleton alterations, as reported in other neurodegenerative disorders^10^, were not previously addressed. Nevertheless, pathways previously described as mediators of TTR neurotoxicity, such as calcium influx and the binding to the receptor for advanced glycation endproducts (RAGE), might signal actin cytoskeleton-related pathways^11–13^.

In this work we aimed to characterize actin cytoskeleton-related pathways involved in FAP-associated axonopathy. We used a FAP *Drosophila* model, expressing human TTRV30M in the fly retina, under the control of GMR promoter, that recapitulates FAP features, namely protein aggregation, similarly to what was previously reported in flies expressing TTRV30M under the pan neuronal *elav* promoter^14^. Using this model, we characterized a novel genetic interaction between TTRV30M and members of Rho GTPase-regulated pathways, major players in actin dynamics and implicated in several neurodegenerative disorders^15^. Considering the high homology and functional conservation observed between the fly and vertebrate neuronal cytoskeleton^16^ we believe that this study can provide valuable insights into the molecular basis of FAP.

## Results

### Characterization of a FAP fly model based on the expression of amyloidogenic TTR in the retina

**Figure 1:**
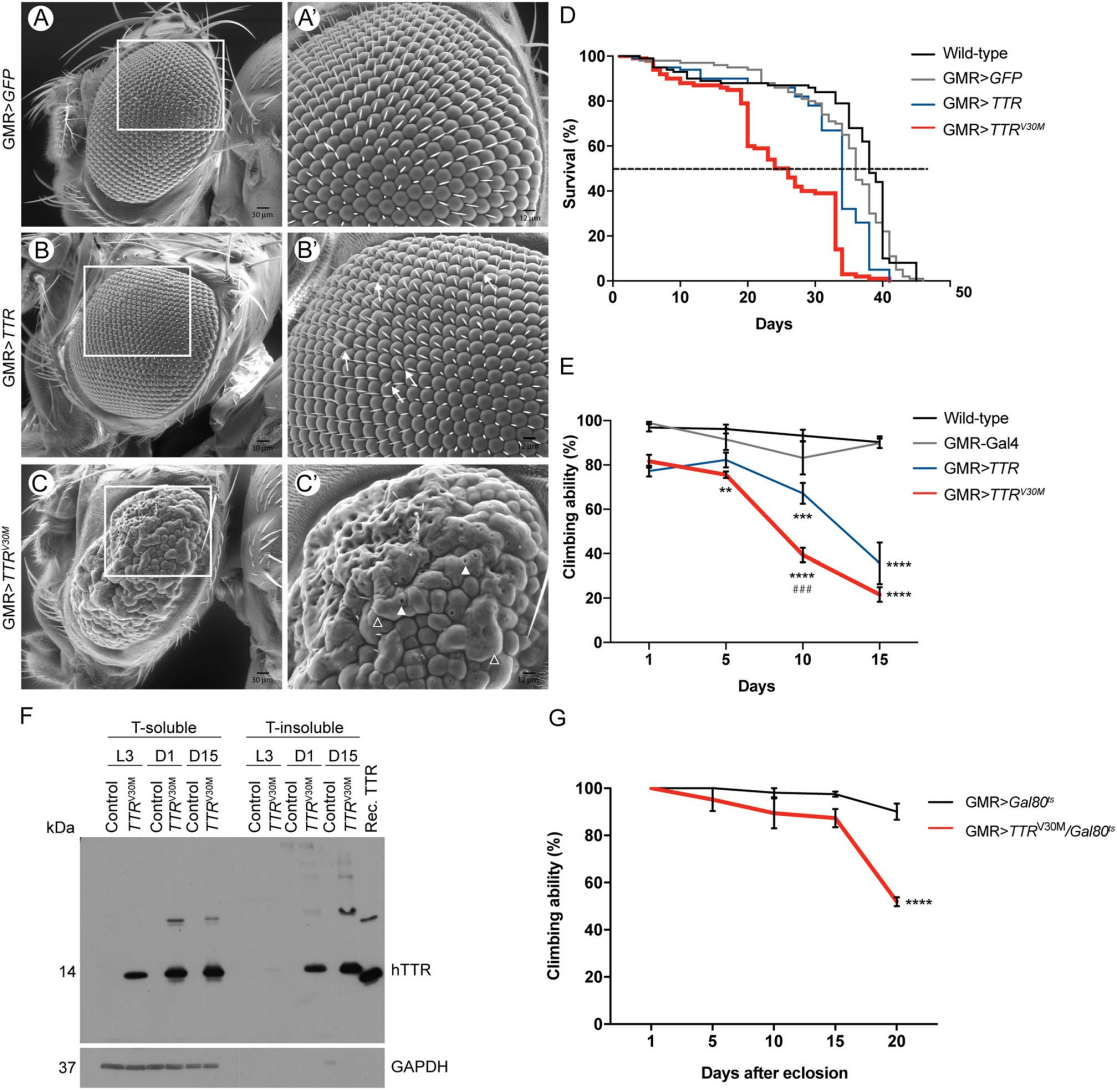
TTRV30M induces neurodegeneration in *Drosophila*. (**A**–**C**) Representative images of scanning electron microscopy (SEM) of fly retinas of the indicated genotypes. (**A**, **A**′) Control flies (GMR>*GFP*) show normal eyes with regular arrangement of ommatidia and bristle orientation. (**B**, **B**′) Flies expressing wild-type TTR display mild irregularities comprising loss or misorientation of bristles (**B**′, white arrows). (**C**, **C**′) Flies overexpressing TTRV30M have smaller eyes, fewer bristles and fused ommatidia (**C**′, white open arrowheads); small holes can be found in the surface of the ommatidia (**C**′, white arrowheads). Scale bar: (**A**–**C**) = 30 μm; (**A**′–**C**′) = 12 μm. (**D**) Lifespan analysis of the genotypes under study. The dashed line indicates the day at which 50% of the flies die. n ≥ 100 flies per genotype. (**E**) Climbing ability of the indicated genotypes. Tests were performed every 5 days during 15 days, after hatching. n = 60 flies per genotype; the results represent the mean ± SEM. Statistical significance determined by Two-Way ANOVA with Turkey’s multiple comparisons test: asterisks (*) significance relative to control flies; cardinals (#) significance of GMR>*TTR*^V30M^ relative to GMR>*TTR*^WT^; ***p < 0.001, ^###^p < 0.001, ****p < 0.0001. (**F**) Western blot analysis of soluble/insoluble TTR in fly extracts. Control (GMR-G4) and GMR>*TTR*^V30M^ flies were analyzed for the presence of TTR in Triton-soluble (T-soluble) and Triton-insoluble (T-insoluble) fractions of the protein extracts. Extracts from larval stages (L3), 1-day old flies (D1) and 15-day old flies (D15) were analyzed. Immunoblot for TTR confirmed the presence of the protein in soluble fractions from larvae and adult flies, and also the presence of TTR in the insoluble fractions of 1- and 15-day old flies. Recombinant TTR (Rec. TTR) was used as positive control. Immunoblot for GAPDH was used as a loading control, confirming the presence of the soluble proteins in the T-soluble fractions. (**G**) Climbing ability of the indicated genotypes. Tests were performed every 5 days during 20 days, after hatching. n ≥ 65 flies per genotype; the results represent the mean ± SEM. Statistical significance determined by Two-Way ANOVA with Sidak's multiple comparisons test.

Previous studies showed that ectopic expression of TTR amyloidogenic mutant isoforms in *Drosophila* induced neurodegeneration^14,17^. In particular, expression of TTRV30M in the developing photoreceptors induces a rough eye phenotype in the adult fly^14^. We used this observation as a starting point to our study, which aimed to analyse whether actin regulators could act as modifiers of the rough eye phenotype of TTRV30M expressing flies. Our analysis showed that flies expressing WT TTR, under the control of GMR-Gal4, had normal size retinas without visible morphological alterations, similar to control flies expressing GFP, as revealed by scanning electron microscopy (Fig. 1A,B). In contrast, ectopic expression of TTRV30M induced smaller eyes, with a severe rough phenotype, characterized by fused and disordered ommatidia, lack of mechanosensory bristles and with foci of necrotic lesions (Fig. 1C). These results illustrate the specific impact of amyloidogenic TTRV30M. We confirmed TTR expression by western blot analysis and observed no differences in TTR protein levels between GMR>*TTR*^V30M^ and GMR>*TTR* flies, discarding the hypothesis that the phenotype observed results from differential expression levels between TTR isoforms (Supplementary Fig. S1A). Next we evaluated the TTR expression pattern in the developing eye-imaginal disc, by comparing with GFP expression within the same genetic background (GMR>*GFP;TTR*^V30M^). Our analysis showed that while GFP expression was restricted to the posterior domain of the developing eye, TTRV30M was detected both in the posterior and anterior domains (Supplementary Fig. S1B,C). These results demonstrate that TTR is secreted to the adjacent tissues (Supplementary Fig. S1C), despite expression driven by GMR promoter being limited to the posterior domain of the eye imaginal disc. Our results are in agreement with data obtained with other TTR-amyloidosis fly models, where TTR was shown to be secreted from photoreceptors and to circulate to other tissues^17^.

Previous studies modeling FAP in *Drosophila* showed that expression of mutant forms of TTR reduced lifespan and impaired climbing ability, signs of neurologic impairment in flies^17^. We recapitulated these analyses using GMR promoter to drive expression of TTRV30M. Our results show that GMR>*TTR*^V30M^ flies have a shorter lifespan when compared to control flies (GMR>*GFP*) (Fig. 1D). The median survival rate of GMR>*TTR*^V30M^ flies was reduced by 35% (from 37 to 24 days) relative to control flies (GMR>*GFP*). In contrast, flies expressing wild-type TTR presented no marked alterations in lifespan having a median survival of 33 days (Fig. 1D). We next evaluated the climbing activity of both genetic backgrounds (Fig. 1E). The climbing ability of control flies was maintained during the course of the experiment, however the climbing ability of TTRV30M expressing flies dramatically decreased to 40% and 22%, by days 10 and 15, respectively. This clearly shows that the phenotype is aggravated with aging. For the same time points, the climbing ability of TTR expressing flies decreased to 67% and 35%, revealing a less accentuated effect of the wild-type TTR on neuronal functions (Fig. 1E).

Following the characterization of the phenotype of the GMR>*TTR*^V30M^ flies, which revealed rough eye phenotype at day 1 and behavior alterations being aggravated with aging, we confirmed protein aggregation in our model. Using western blot analysis of soluble and insoluble fractions of head extracts from GMR>*TTR*^V30M^ flies, we observed the presence of insoluble TTRV30M aggregates in 1 and 15-days old flies, which were increased with age (Fig. 1F). In TTRV30M larval brains no insoluble aggregates were detected (Fig. 1F). However, considering the amount of aggregated protein at day 1, we cannot exclude the formation of soluble toxic oligomers at this stage.

TTRV30M-associated neurotoxicity is also detected when expression is limited to adult stages. Using the Gal80^ts^ system we promoted TTRV30M expression in the retina during adult stage, and assayed flies climbing performance. Our analysis shows that TTRV30M expressing flies show increased climbing defects by day 20, relative to controls (Fig. 1G). Altogether, these findings highlight the specific neurotoxicity of TTRV30M and validate the use of GMR>*TTR*^V30M^ flies as a relevant model to use in genetic screens aiming at identifying modifiers of TTRV30M induced- phenotype.

### TTRV30M induces actin cytoskeleton alterations in photoreceptor axons

**Figure 2:**
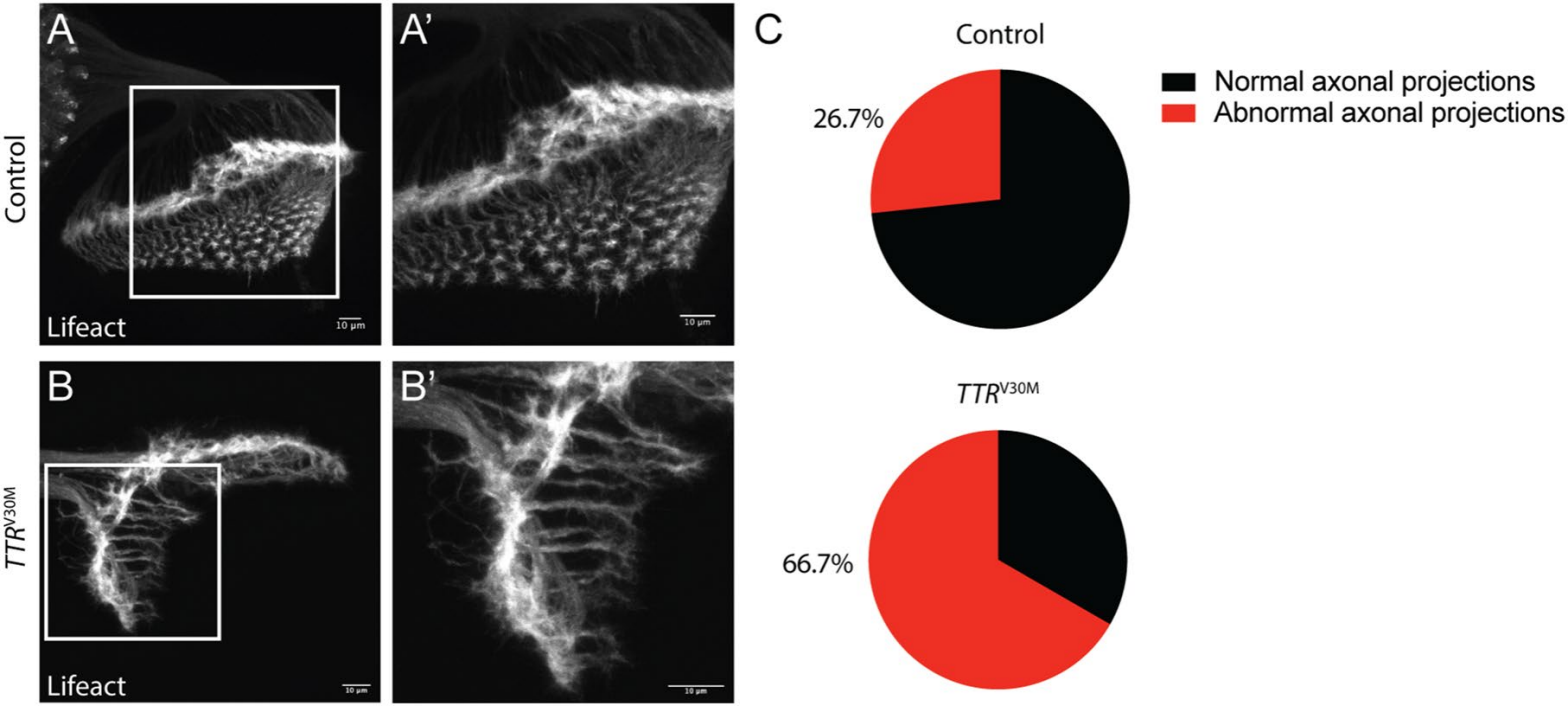
TTRV30M expression in the retina induces axonal projections defects. (**A**, **B**) The actin reporter UAS-Lifeact-ruby was used to evaluate axonal projections from 3rd instar eye-imaginal discs into larval brains. (**A**, **A**′) In GMR>*Lifeact* larvae (control) retinal axons project into the lamina and medulla layers in an organized manner. (**A**′) High magnification of (**A**) showing the normal array of the axonal projections and growth cones in the medulla layer. (**B**, **B**′) GMR>*Lifeact;TTR*^V30M^ expressing larvae (*TTR*^*V30M*^) exhibit abnormal axonal projections. (**B**′) High magnification of (**B**) showing a disruption of the typical star-shaped structure of the growth cones. Scale bar: 10 μm. (**C**) Pie chart representation of the percentage of eye imaginal discs that display axonal abnormalities, as determined by qualitative analysis. GMR>*Lifeact* (Control, n = 15); GMR>*Lifeact;TTR*^V30M^ (*TTR*^*V30M*^, n = 12).

As in many other studies using *Drosophila* to model neurodegeneration, we found that TTRV30M expressing flies have a severe rough eye phenotype by day 1. Actually, this suggests that amyloidogenic TTR might have an impact on neuronal morphogenesis. As such, we evaluated the cytoskeleton organization of photoreceptor axons at the third instar larval stage by crossing GMR>*TTR*^V30M^ flies with Lifeact expressing flies, to label F-actin. We observed that contrarily to control flies (Fig. 2A), TTRV30M expressing larvae exhibited defects in axonal projections. In 8% of the TTRV30M expressing larvae axons failed to project into the brain area although photoreceptors differentiated within the eye disc (data not shown); in the remaining 92%, axons extended projections into the brain region, however the majority (66.7%) in a highly disorganized manner (Fig. 2B,C). The abnormal projections observed in TTRV30M expressing larvae comprised axons with misguided orientation, with some of them failing to reach the proper target layer and apparently growing in an opposite direction. Within the medulla, axons also projected in an irregular manner, failing to reach their target, and some medulla regions lacked axonal projections (Fig. 2B). In addition, growth cones of projecting axons lacked the normal spread distribution of filopodia and lamellipodia actin structure, suggesting altered actin organization (Fig. 2B′).

Additionally, we analyzed axonal projections of PDF expressing neurons in GMR>*TTR*^V30M^ flies (Supplementary Fig. S2). Abnormal axonal arborization was detected in larvae, that was clearly aggravated in adult stage (Supplementary Fig. S2C,D). The area of PDF expressing neurons is larger in GMR>*TTR*^V30M^ adult flies (Supplementary Fig. S2D,E). We also observed a disorganized spatial arrangement of cell bodies of PDF expressing neurons (Supplementary Fig. S2D′). These results indicate that TTRV30M toxicity influences the cytoskeleton, impacting on axonal projection and organization.

### TTR interacts genetically with members of Rho GTPase signaling pathways

**Figure 3:**
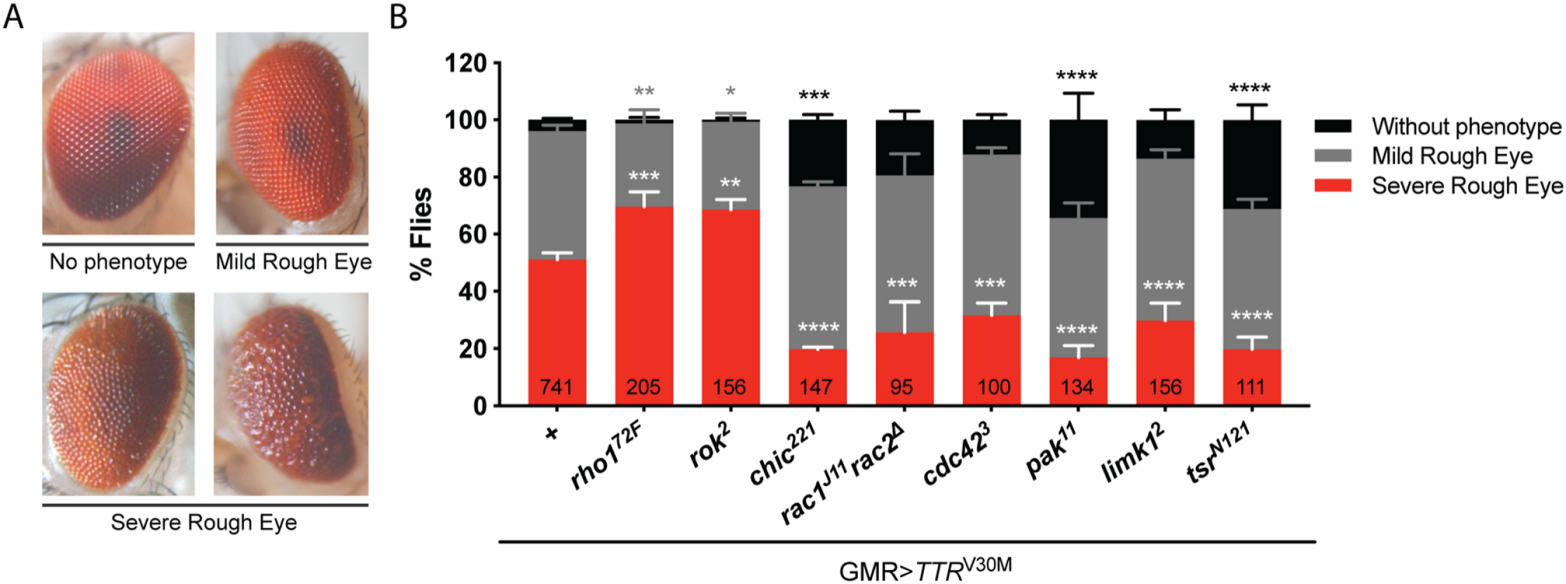
Candidate genetic screen with members of Rho GTPase signaling pathways. (**A**) Representative pictures of the different phenotypes observed in adult retinas and the corresponding categories: no phenotype, mild rough eye (flies with a minor or marked disorganization of the compound eye), severe rough eye (flies with highly disorganized structure of the compound eye and with bigger ommatidia). (**B**) Percentage of flies in each eye phenotype category. Flies expressing TTRV30M and lacking one copy of the gene indicated were analyzed for suppression or enhancement of the TTRV30M-associated rough eye phenotype (first column, GMR>*TTR*^V30M^ crossed with wild-type flies). At least 3 independent experiments (total number of flies indicated in the respective column) were performed and only females were evaluated for each genotype. The results were plotted as mean ± SEM. Statistical significance determined by Two-Way ANOVA with Dunnett’s multiple comparison test *p < 0.05, **p < 0.01, ***p < 0.001, ****p < 0.0001.

The retina phenotype associated with TTRV30M expression was the basis for a genetic screen aimed to identify actin cytoskeleton regulators that might represent novel TTR interacting molecules. We evaluated TTR interactions using null alleles for each gene under test. The use of mutant alleles has the advantage of avoiding the fluctuating levels of RNAi-mediated downregulation and possible off-target effects. For control, GMR>*TTR*^V30M^ homozygous flies were crossed with wild-type flies so that there is only one copy of the UAS-*TTR*^V30M^ transgene in the background, similar to what is obtained after crossing GMR>*TTR*^V30M^ with the different mutant alleles. Heterozygous flies for mutations in each of these genes do not show eye phenotype. Our screen was focused on members of the Rho GTPase family, major regulators of actin dynamics and implicated in several neurodegenerative disorders^15^. We classified as suppressors or enhancers the interactions leading to a reduction or increase in the percentage of flies with severe rough eye phenotype (Fig. 3A), respectively. We observed that haploinsufficiency for *rho1* and *rok* induced an increase in the percentage of flies with severe rough eye phenotype (Fig. 3B). These results imply that downregulation of the Rho1-Rok pathway acts as an enhancer of TTRV30M-induced neurotoxicity. Haploinsufficiency for *rac1/2* and *cdc42* induced a suppression of the rough eye phenotype. In agreement with these observations, decreasing the levels of Pak and LIMK, downstream effectors of Rac1 and Cdc42, also suppressed the TTRV30M-induced phenotype (Fig. 3B). Additionally, haploinsufficiency for *twinstar* (*tsr*), the *Drosophila* homolog for cofilin/actin depolymerization factor (ADF), and *chickadee* (*chic*), the *Drosophila* homolog of profilin, two actin binding proteins, crucial for actin dynamics regulated by Rho GTPase signaling, led to suppression of the TTRV30M-induced phenotype (Fig. 3B). In this study and through the identification of modifiers of TTRV30M-induced rough eye phenotype, we uncovered Rho1 and Rok as enhancers, and Rac1/2, Cdc42, Pak, LIMK, Twinstar and Chickadee as suppressors, highlighting a novel genetic interaction between TTRV30M and Rho GTPase signaling pathways with impact on actin dynamics regulation.

### TTRV30M leads to axonal cytoskeleton alterations via Rho GTPases

**Figure 4:**
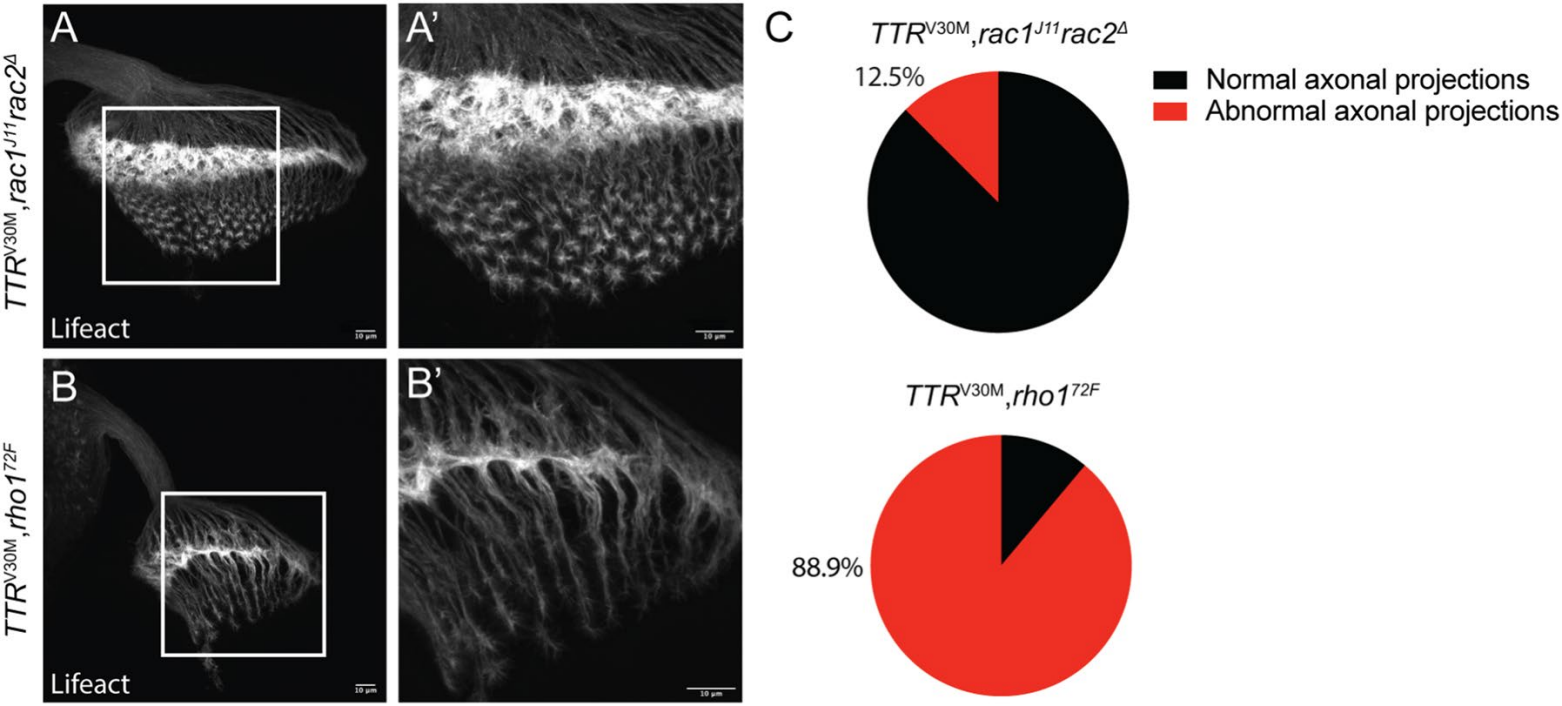
Rac1/2 and Rho1 are modifiers of TTRV30M-induced axonal defects. (**A**, **B**) Analysis of axonal projections in 3rd instar larvae brains expressing the actin reporter, UAS-Lifeact-ruby, under the GMR promoter. Scale bar: 10 μm. (**A**, **A**′) Haploinsufficiency for *rac1, rac2* in TTRV30M expressing larvae (*TTR*^*V30M*^*,rac1*^*J*11^*rac2*^*Δ*^*)* rescues the axonal defects promoted by TTRV30M. (**B**, **B**′) Haploinsufficiency for *rho1* in TTRV30M expressing larvae (*TTR*^*V30M*^*, rho1*^*72F*^), enhances the axonal defects induced by TTRV30M. (**C**) Pie chart representation of the percentage of eye imaginal discs that display axonal abnormalities, as determined by qualitative analysis, in the following genotypes GMR>*Lifeact;TTR*^V30M^/*rac1*^J11^*rac2*^Δ^ (*TTR*^*V30M*^* ,rac1*^*J*11^*rac2*^*Δ*^*,* n = 8); GMR>*Lifeact/rho1*^72F^*;TTR*^V30M^ (*TTR*^*V30M*^*, rho1*^*72F*^*,* n = 9).

We observed that TTRV30M disturbs axonal projection of photoreceptors (Fig. 2B). We next asked if the modifiers of TTRV30M-associated phenotype played a role in this axonal phenotype. Accordingly, we selected a suppressor (*rac1; rac2* double mutation) and an enhancer (*rho1*) and evaluated the impact of haploinsufficiency of these genes in the axonal projections of TTRV30M expressing larvae. We observed that removal of one copy of both *rac1* and *rac2* (*rac1*^J11^*rac2*^∆^) reverted the axonal projection phenotype (Fig. 4A,C), with only 12.5% of the larvae showing abnormal axonal projections (Fig. 4C), which is in agreement with the results observed with adult retinas. In contrast, haploinsufficiency for *rho1* led to an increase, to 88.9%, in the percentage of larvae with abnormal axonal projections, in agreement with *rho1* downregulation acting as an enhancer of TTR-mediated phenotypes (Fig. 4B,C). Of note, in this genetic background the growth cones appear even more disrupted, exhibiting a blunt-ended morphology (Fig. 4B′). Axonal projections from heterozygous larvae for *rac1*^J11^*rac2*^∆^ and *rho1*^72F^ are indistinguishable from control larvae (GMR>*Lifeact*) (Supplementary Fig. S3A–C). These results show that TTRV30M interacts genetically with Rac1/2 and Rho1 to modify axonal behavior and actin organization.

## Discussion

In this work, taking advantage of a *Drosophila* FAP model^14^ we unraveled novel genetic interactions between TTRV30M and members of the Rho GTPase signaling pathways, associated with neurotoxicity. Additionally, our work describes the first genetic screen towards the identification of modifiers of TTRV30M-mediated neurotoxicity, adding a valuable model for the identification of novel players mediating neurodegeneration in FAP.

We used a fly model based on the expression of TTRV30M, the most common TTR mutation associated with FAP^14^, in photoreceptors. This model recapitulated the previously described rough eye phenotype, a critical tool to use as readout in this study. We observed that flies expressing wild-type TTR have a normal adult retina, although protein levels were similar to the ones in TTRV30M expressing flies, confirming the specific effect of the amyloidogenic mutant. In addition to the eye phenotype, we analysed lifespan and climbing activity of TTRV30M expressing flies. We observed that flies expressing TTRV30M displayed reduced lifespan and impaired climbing activity that was aggravated with aging, reflecting the progressive deleterious effect of TTRV30M. These degenerative phenotypes reflect TTR neurotoxicity and can be explained by the finding that TTRV30M is secreted from retina expressing cells. Similar results were observed with other amyloidogenic mutants, TTRL55P and TTRV14N/V16E when expressed under the same promoter^17^. According to our study, TTRV30M insoluble aggregates are detected in 1-day old flies. Since toxicity correlates mainly with soluble aggregation states, we propose that soluble TTR expressed in the retina is secreted to the hemolymph where it forms soluble aggregates inducing neurotoxicity in adjacent tissues like the brain, leading to impaired neuronal function. These results are in line with what happens in vertebrates where TTR is synthesized in liver and choroid plexus and secreted to plasma and CSF, respectively, being able to reach other tissues, as is the case of the sciatic nerve^8^. Our characterisation validated TTRV30M expressing flies as a suitable model to study TTRV30M-induced neurotoxicity, and adequate to use in genetic screens.

Our analysis showed that the TTRV30M-associated phenotype arises during larval development, as indicated by the defective axonal projections observed in TTRV30M-expressing larvae. Although this model does not exactly recapitulate FAP, as patients are asymptomatic during the development and maturation of the nervous system, the occurrence of developmental associated defects underlying disease has not been addressed to date.

Considering the impact of cytoskeleton dysfunction on neurodegenerative disorders^10^, and the fact that neurotoxicity in FAP is mediated by interaction of TTR aggregates with the RAGE receptor^12^, which was previously shown to be involved in the control of actin cytoskeleton remodelling^13^, we performed a small-scale guided genetic screen focused on Rho GTPase signaling pathways to determine if they could act as modifiers of TTRV30M-induced neurotoxicity. Our analysis shows that downregulation of the Rho1-Rok pathway enhances TTRV30M-induced rough eye phenotype, and axonal projection defects while the downregulation of the Rac/Cdc42/Pak/LIMK pathway has the opposite effect, and acts as a suppressor of both phenotypes. RhoA/ROCK signaling has been implicated in several neurodegenerative disorders, and inactivation of the pathway, mainly through the use of ROCK inhibitors, promoted a beneficial impact on those disorders^10^. Contrarily, our results point to a negative effect of Rho1 and Rok downregulation on TTRV30M-induced neurotoxicity. Downregulation of RhoA/ROCK leads to a decrease in phosphorylation of profilin impacting on the activation of the protein. This suggests that in our settings where downregulation of Rho1/Rok aggravated TTRV30M-induced phenotype increased levels of active profilin should induce a similar effect. Accordingly, we observed a suppression of the TTRV30M-induced rough eye phenotype with the downregulation of chickadee, the *Drosophila* homolog of profilin. Importantly, profilin mutations were related with neurodegeneration in Amyotrophic lateral sclerosis (ALS)^18^.

The impact of Rac on neurodegeneration is still unclear. It was shown that both upregulation and downregulation of Rac1/2 led to amelioration on different neurodegenerative disorders^19^, highlighting a disease and context specific impact. Studies in *Drosophila* neurons demonstrated that activation of the Trio GEF-Rac/Cdc42/Pak/LIMK pathway lead to inhibition of axonal growth, through LIMK-mediated cofilin phosphorylation and inactivation and consequently dysregulation of actin dynamics^20^. Our data shows that downregulation of Rac1/2, Cdc42, Pak and LIMK suppress TTRV30M-induced defects, in accordance with the referred study^20^. Moreover, decreased levels of twinstar, the *Drosophila* homolog of cofilin, a LIMK substrate, also suppressed the TTRV30M-induced rough eye phenotype.

In agreement with an interaction between TTRV30M and members of the Rho GTPase family, the disorganization of the actin cytoskeleton in photoreceptor axons of TTRV30M-expressing larvae was modified by Rho1 and Rac. We observed an antagonistic effect of Rac/Cdc42 and Rho1 on the modification of TTRV30M-induced phenotype. This opposite effect might be a result from the inter-regulation between the members of the Rho GTPases family. In fact, it has been shown that RhoA and Rac can inhibit each other, mainly by acting on the regulation of GEFs and GAPs (GTPase-activating proteins)^21^.

In conclusion, our data uncovered Rho GTPase signaling pathways as novel players in the molecular mechanisms through which TTRV30M potentially induces neurotoxicity. Most likely this is mediated through signaling, as we failed to detect a direct interaction between TTRV30M and Rho1, and Rac1 (Supplementary Fig. S4). Additionally, our observations suggest that Rho GTPase pathway modulators might have a beneficial impact on FAP. In the case of Alzheimer's Disease (AD), the use of ROCK inhibitors to modulate RhoA was shown to impact on disease phenotype. Moreover, NSC23766 and some nonsteroidal anti-inflammatory drugs (NSAIDs) were suggested as beneficial therapeutic options targeting Rac1 and Cdc42^22^. Concerning our data, Rac1 and Cdc42 inhibitors could be a suitable option for TTR-induced neurodegeneration. Nevertheless, future research will address the significance of Rho GTPase signaling pathways in FAP mouse models, that will contribute to increase the knowledge on the pathogenesis of the disease.

## Materials and methods

### Fly strains and genetics

The fly strains used in this study are described in Supplementary Table S1. Fly stocks and crosses were maintained in standard growth media, at 25 °C, unless stated otherwise. The Gal4/UAS system was used to drive expression of human TTR transgenes in the fly retina, under the control of GMR-Gal4^23^. Transgenic flies enabling the expression of human wild type (WT) TTR (UAS-*TTR*) and a mutant and strong amyloidogenic form TTRV30M (UAS-*TTR*^V30M^-HA) were previously reported^14,17^. Standard genetic techniques were used to generate the different genetic backgrounds in this study (Supplementary Table S1).

For the genetic interaction screen, GMR>*TTR*^V30M^ females were crossed with males from the mutant allele under test. To test interactions with genes located on the X chromosome males GMR>*TTR*^V30M^ were used in the cross. For control GMR>*TTR*^V30M^ homozygous flies were crossed with WT flies, so that there is only one copy of the UAS-*TTR*^V30M^ transgene in the background, similar to what is obtained after crossing GMR>*TTR*^V30M^ with the different mutant alleles. Crosses were performed at 25 °C and eggs were transferred and let to develop at 29 °C to enable maximum expression levels of UAS-transgenes. The retina phenotype of 1-day old flies (F1 progeny) was analyzed and flies were scored into three categories: no phenotype, mild rough eye and severe rough eye (Fig. 2A). Results were plotted as the percentage of flies in each category. Data analysis was performed with Two-Way ANOVA with Dunnett’s multiple comparisons test. Adult retina phenotype was imaged in a stereomicroscope Stemi 2000 Zeiss equipped with a Nikon Digital SMZ 1500 camera, with a 50 × magnification.

### Lifespan assay

Recently hatched flies were allowed to mate for 2 days and separated by gender into groups of 20 flies, in a total of 100 flies per genotype. Flies were maintained at 29 °C throughout the assay. Flies were transferred into fresh vials every 2–3 days and the dead flies were scored. The survival curves were plotted as the percentage of live flies at each time point.

### Climbing assay

Recently hatched flies were allowed to mate for 2 days and separated by gender into groups of 20 flies, in a total of 60 flies per genotype. Flies were maintained at 29 °C throughout the assay. To perform the climbing assay, flies were gently shaken to the bottom of the tube and allowed to climb for 20 s. The number of flies that were able to climb up to 5 cm was counted and expressed as a percentage, which refers to climbing ability. Each set of flies was tested three times at each time point (1, 5, 10, 15, and 20 days after hatching). Results are plotted as mean ± SEM. Data analysis was performed with Two-Way ANOVA with Tukey's multiple comparisons test.

### Extract preparation for protein analysis

For analysis of TTR expression levels six heads from1-day old flies, per genotype, were homogenized in 20 μL of Laemmli Sample Buffer (50 mM Tris–HCl pH 6.8, 2% SDS, 100 mM β-mercaptoethanol, 10% glycerol and 0.1% bromophenol blue) and snap frozen. Prior to SDS-PAGE analysis samples were heated at 100 °C for 5 min and centrifuged at 13,000 rpm for 5 min.

For analysis of soluble and insoluble fractions thirty adult fly heads, and fifty third instar larval brain and imaginal discs, were used per genotype. Tissue homogenization was performed in 100 μL of extraction buffer (PBS with 1% triton X-100 (Sigma-Aldrich), protease inhibitors (Protease Inhibitor Mix GE Healthcare, 80-6501-23, 1:100) and sodium orthovanadate 1 mM) for 30 s. Extracts were centrifuged at 15,000*g* for 10 min at 4 °C and supernatant was collected as the Triton-soluble fraction. The pellet was washed 3 times with extraction buffer and resuspended in 100 μL of 50 mM Tris pH 7.5 with 4% SDS and protease inhibitors followed by sonication. Extracts were centrifuged at 15,000*g* for 10 min at 4 °C and supernatant was collected as the Triton-insoluble fraction. SDS-PAGE and western blot analysis were performed as described below.

### Immunoprecipitation

Two hundred flies per genotype were collected, frozen with liquid nitrogen and vortexed for 15 s to separate fly heads. Fly heads were homogenized in PBS with 0.3% triton X-100 (Sigma-Aldrich), protease inhibitors (EDTA-free Protease Inhibitor Cocktail Sigma-Aldrich 1:100) and sodium orthovanadate 1 mM). Head lysates were centrifuged at 10,000 rpm for 20 min at 4 °C, and the supernatant was pre-cleared prior to use in immunoprecipitation. To pre-clear lysates, 250 μg of head lysate were incubated with 25 μL of magnetic beads (Dynabeads™ Protein G, Novex) overnight. Pre-cleared head lysate was incubated with bead-antibody (Ab) complex overnight at 4 °C. 2 μg of anti-human TTR (A0002, DAKO) coupled to 25 μL of magnetic beads were used. Magnetic beads—pelleted TTR—protein complexes were eluted with 2 × Laemmli Sample Buffer. SDS-PAGE and western blot analysis were performed as described below.

### Western blot

Protein extracts were applied on 15% SDS-PAGE gels and transferred to a 0.45 μm nitrocellulose membrane (Amersham). Membrane was blocked in 5% non-fat dry milk in TBS-T [TBS with 0.05% Tween (Sigma-Aldrich)] for 1 h, prior to overnight incubation at 4 °C with primary antibodies: rabbit anti-hTTR (A0002, DAKO, 1:1,000), mouse anti-α-Tubulin (T5168, clone B512, Sigma-Aldrich, 1:100,000), mouse anti-GAPDH (G-9, sc365062, Santacruz, 1:500), mouse anti-Rho1 (P1D9, Developmental Studies Hybridoma Bank, 1:250), mouse anti-Rac1 (23A8, ab33186 Abcam, 1:2000) in blocking buffer. Incubation with horseradish peroxidase-labeled secondary antibodies was performed for 1 h at room temperature in blocking buffer. Blots were developed using the enhanced chemiluminescence western blot substrate (Bio-Rad).

### Immunohistochemistry

Third instar larvae were collected and dissected in ice cold PBS. Larval eye-antenna imaginal discs and brain were dissected, fixed in 3.7% formaldehyde in PBS for 20 min and mounted in 50% glycerol in PBS. Larval samples fixed and processed for immunohistochemistry were washed three times in PBT 0.3% [1 × PBS + 0.3% Triton X-100 (Sigma-Aldrich)] and incubated with primary antibodies: rabbit anti-HA (Abcam Ab9110, 1:50), rat anti-DCAD2 (Developmental Studies Hybridoma Bank, 1:100), mouse anti-PDF (C7, Developmental Studies Hybridoma Bank, 1:100) in PBT 0.3%, overnight at 4 °C. Secondary antibodies (Alexa anti-rabbit 568 (A10042), Alexa anti-rat 647 (A21247), Alexa anti-mouse 488 (A21202) ThermoFisher Scientific) were diluted in PBT 0.1% (1 × PBS + 0.1% Triton X-100) and incubated for 3 h at room temperature. Discs were mounted in 50% glycerol in PBS. For F-actin labeling, Lifeact expressing larvae were imaged using an inverted laser scanning confocal microscope (Leica TCS SP5II) with a 63 × objective. Image stacks with 0.3–0.5 μm of z-step size were acquired of each imaginal disc and brain and z-stacks of interest were projected using Fiji software. The percentage of eye-imaginal discs with abnormal axonal projections was determined by qualitative analysis of the images. At least 7 imaginal discs were analyzed per genotype.

Brains from adult flies (1 day old) were dissected in cold PBS and fixed in 3.7% formaldehyde in PBS for 30 min. Samples were washed three times in PBT 0.5% [1 × PBS + 0.5% Triton X-100 (Sigma-Aldrich)] and incubated in blocking buffer (PBT 0.5% + 0.5% BSA + 0.5% FBS) for 1 h 30 min at room temperature. Incubation with primary antibodies: mouse anti-PDF (C7, Developmental Studies Hybridoma Bank, 1:100) was performed in blocking buffer overnight at 4 °C. Secondary antibody (Alexa anti-mouse 488 (A21202) ThermoFisher Scientific) was diluted in PBT 0.5% and incubated for 3 h at room temperature. Tissues were mounted in Fluoromount-G™ Mounting Medium (ThermoFisher Scientific). Whole-mount adult brain samples were imaged using an inverted laser scanning confocal microscope (Leica TCS SP5II) with a 40 × or 63 × objectives. Image stacks with 0.7–1 μm of z-step size were acquired of each sample and the z-stacks of interest were projected using Fiji software.

Whole-mount adult brains immunostained for PDF-positive neurons were imaged as previously described and z-stacks projected into a single image. Quantification was performed in grayscale images and the areas of the axonal arborization and of the brain hemisphere were measured. For that, the polygon selection tool of the Fiji software was used and the limits of the axonal projection and the brain hemisphere were drawn and the area calculated. The normalized area represents the area of the PDF axonal projections divided by the area of the respective brain hemisphere. 7 brains from control (GMR-G4) flies and 11 brains from GMR>*TTR*^V30M^ flies were analysed.

### Scanning electron microscopy (SEM)

For SEM analysis, 1-day old flies were dehydrated through incubation in ethanol series (25%, 50%, 75%, 100%), incubated with hexamethyldisilazane (HMDS, Sigma), air-dried, and mounted in SEM stubs and coated with Au/Pd thin film, by sputtering using the SPI Module Sputter Coater equipment. Imaging was performed using a High resolution (Schottky) Environmental Scanning Electron Microscope with X-ray microanalysis and Electron Backscattered Diffraction analysis: Quanta 400 FEG ESEM/EDAX Genesis X4M. The images were acquired with 400 × and 1,000 × magnification.

### Statistical analysis

The statistical analysis was performed with GraphPad Prism software version 8. The assays with more than one experiment are represented as mean ± SEM (standard error of the mean). Data comparison between groups was performed using Two-Way ANOVA with Dunnett’s multiple comparisons test, with Tukey's multiple comparisons test or Sidak’s multiple comparison test, as referred in each experiment. p < 0.05 was considered statistically significant.

## Supplementary information

Supplementary Information.

## Data Availability

Raw data generated during this study is available upon request.
