# Supplementary material for: Transthyretin interacts with actin regulators in a Drosophila model of familial amyloid polyneuropathy
Source: Sci Rep. 2020 Aug 12;10:13596. doi: 10.1038/s41598-020-70377-4 (PMC7423984; doi:10.1038/s41598-020-70377-4)
Supplement: Supplementary file 1 — Supplementary Information. [file 41598_2020_70377_MOESM1_ESM.pdf]

Transthyretin Interacts with Actin Regulators in a *Drosophila* model of Familial Amyloid Polyneuropathy

Marina I. Oliveira da Silva[1,2], Carla S. Lopes[3*], Márcia A. Liz[2*]

Supplementary Information

Supplementary Figures

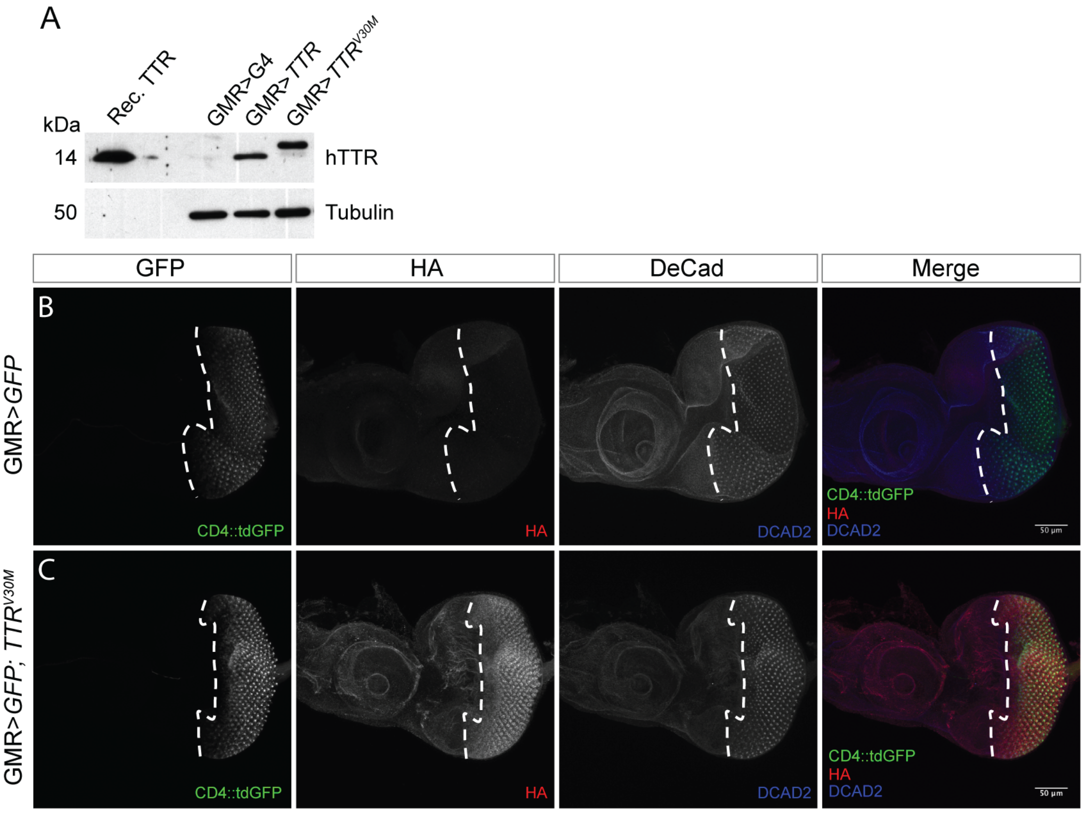

Supplementary Figure S1. Expression of TTR in the fly developing eye and retina. (A) Western blot of 6 fly head extracts from 1-day-old flies of the following genotypes: GMR>*G4*, GMR>*TTR*, GMR>*TTR*^{V30M}. The presence of a HA tag on TTRV30M results in a band with higher molecular weight. Recombinant TTR (Rec. TTR) was used as positive control. Tubulin was used as loading control. **(B, C)** Immunohistochemistry on 3rd instar eye-imaginal discs of the following genotypes: GMR>*GFP* (B), GMR>*GFP*;*TTR*^{V30M} (C). Expression of CD4::tdGFP (green) was used to define the domain of expression of GMR-Gal4 promoter. HA (red) was used to detect TTRV30M. DECAD2 staining (blue) was used to visualize epithelia morphology. Scale bar: 50µm.

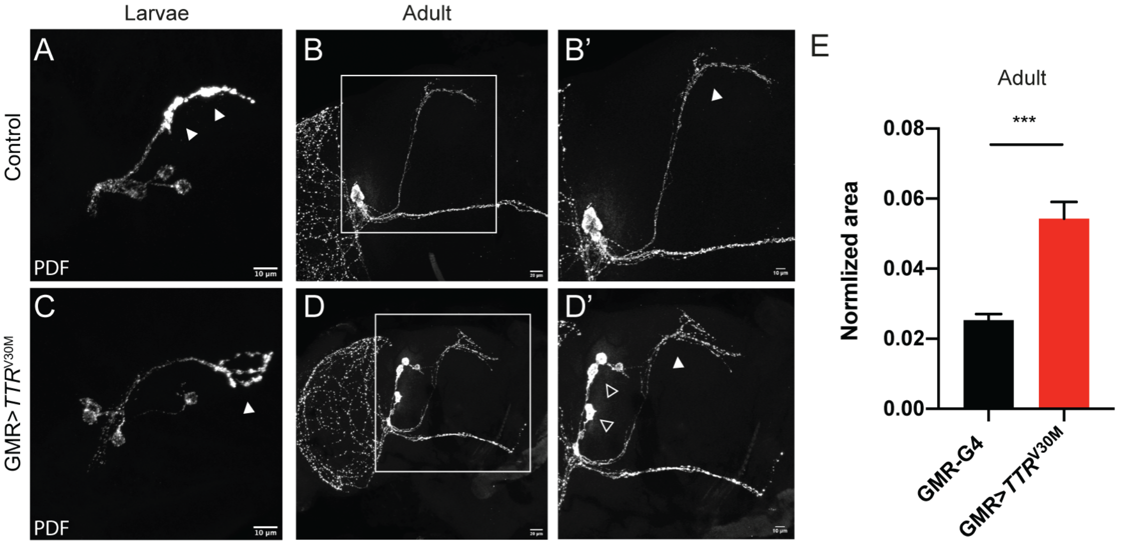

Supplementary Figure S2. TTRV30M expression in photoreceptors affects axonal arborization of PDF neurons (A, C) Immunohistochemistry of 3rd instar larval brains of control (GMR-G4) and GMR-*TTR*^{V30M}. Scale bar: 10µm **(B)** Immunohistochemistry on brains from 1-day old control flies (GMR-G4). Scale bar: 20µm **(B')** High magnification of (B) showing the normal arrangement of axonal arborization (white arrowheads). Scale bar: 10µm **(D)** Immunohistochemistry of PDF expressing neurons in adult brains from 1-day old GMR>*TTR*^{V30M} flies. Scale bar: 20µm **(D')** High magnification of (D) showing increased axonal arborization (white arrowheads) and the disorganized spatial arrangement of cell bodies (open arrowheads). Scale bar: 10µm **(E)** Quantification of the area occupied by the axonal arborization of the PDF-positive neurons normalized by the respective area of the brain hemisphere, in 1-day old flies. Statistical significance determined by Two-Way ANOVA with Sidak's multiple comparisons test.

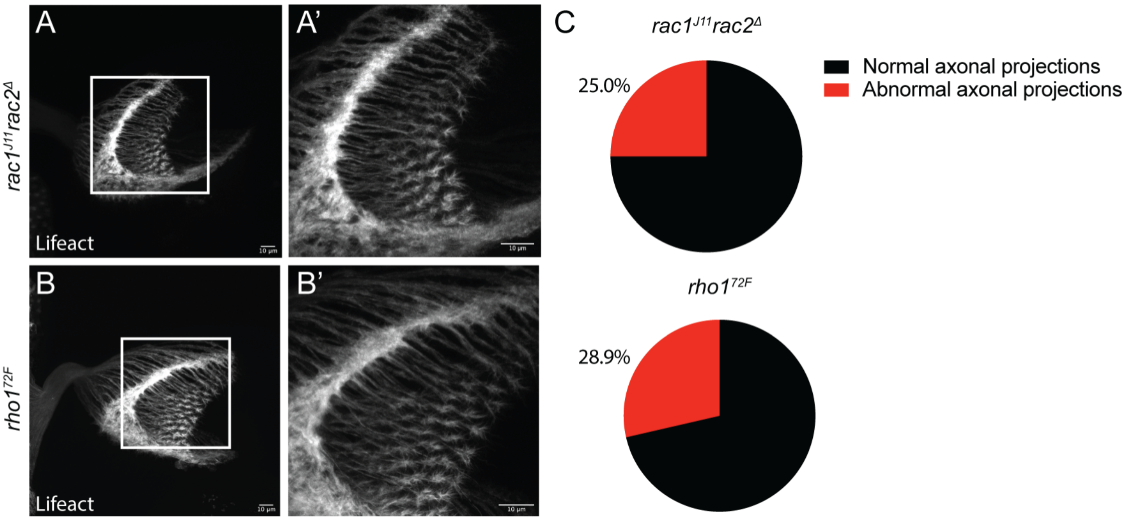

Supplementary Figure S3. Haploinsufficiency for *rho1* or *rac1*$^{\mathbf{J11}}$*rac2*$^{\Delta}$ does not affect axonal projections organization. (A, B) Analysis of axonal projections in 3rd instar larvae brains expressing the actin reporter, UAS-Lifeact-ruby, under the GMR promoter. Scale bar: 10µm. **(A, A')** GMR>*Lifeact;rac1*^{J11}*rac2*$^{\Delta}$ (*rac1, rac2*) larvae do not display defects in axonal projections. **(B, B')** Axonal defects were not detected in GMR>*Lifeact/rho1*^{72F} (*rho1*^{72F}) larvae. **(C)** Pie chart representation of the percentage of larvae with abnormal axonal projections evaluated qualitatively. GMR>*Lifeact;rac1*^{J11}*rac2*$^{\Delta}$ (*rac1*^{J11}*rac2*$^{\Delta}$, n=8); GMR>*Lifeact/rho1*^{72F} (*rho1*^{72F}, n=7).

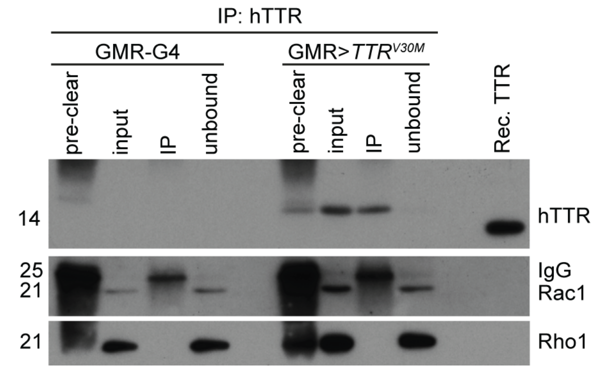

Supplementary Figure S4. Rac1 and Rho1 do not co-immunoprecipitate with TTRV30M. Co-immunoprecipitation of Rac1and Rho1 using the anti-human TTR antibody, in extracts from control (GMR-G4) and GMR>*TTR*^{V30M} adult fly heads. Lanes: Pre-clear, pellet from extract beads incubation showing unspecific binding to the magnetic beads. Input, pre-cleared extract used for the immunoprecipitation assay. IP, proteins precipitated with anti-TTR-beads complex. Unbound, extract not bound to anti-TTR-beads complex. TTR is present in the input and recovered in the IP fraction. Rac1 and Rho1 are present in the input but are not recovered in the IP fraction.

Full-length blots Figure S1A

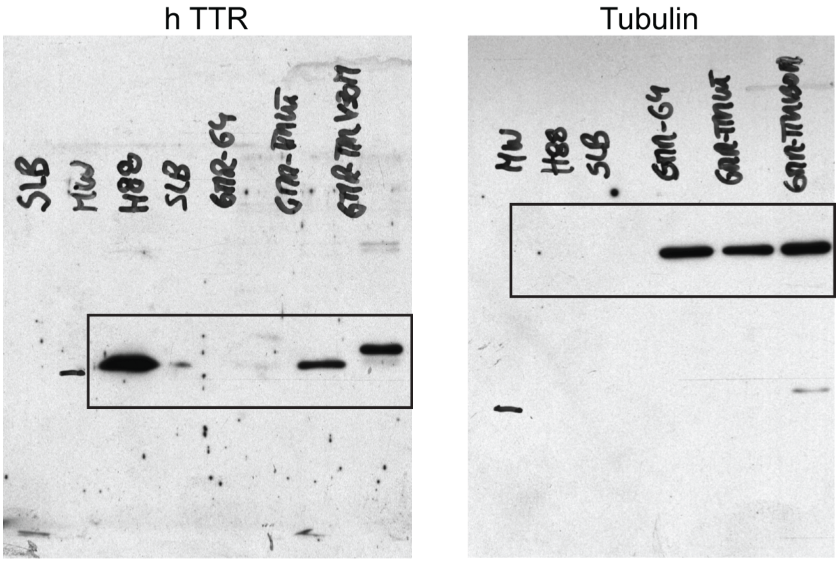

Full-length blots Figure S4

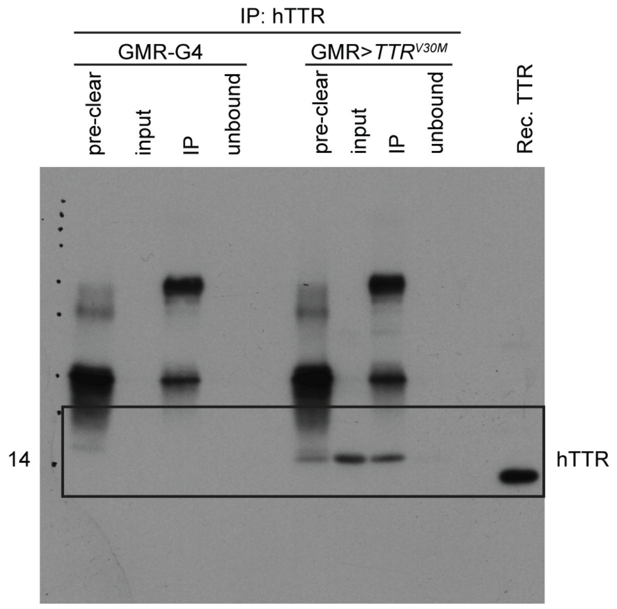

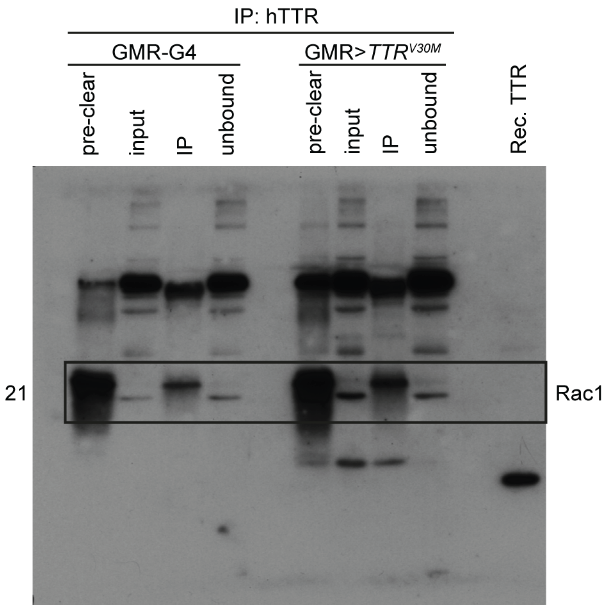

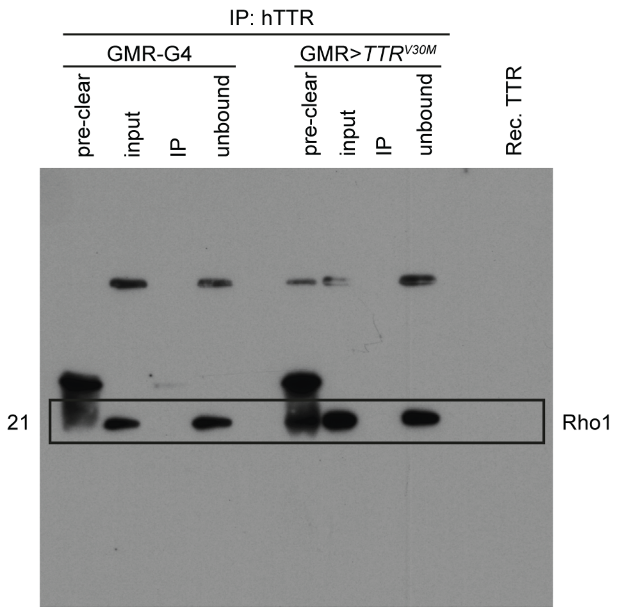

Supplementary Table

Table S1. List of fly strains used and generated in this study with respective genotype and source.

Stock	Genotype	Source
**Wild-type**	w^{1118}	in-house
**GMR-*GAL4***	GMR-Gal4/GMR-Gal4	(Hay *et al.*, 1997)
**Gal80^{ts}**	P{tubP-GAL80[ts]}2	Bloomington 7017
**UAS-*Lifeact***	y^1 w*; P{UAS-Lifeact-Ruby}VIE-19A	Bloomington 35545
**UAS-*Lifeact***	w*; sna^{Sco}/CyO; P{UASt-Lifeact-RFP}3	Bloomington 58362
**UAS-*mCD4-GFP***	w^{1118};PBac{UAS-CD4-tdGFP}VK00033	Bloomington 35836
**UAS-*TTR***	w^{1118}; P{UAS-TTR^{wt}}	(Pokrzywa *et al.*, 2007)
**UAS-*TTR^{V30M}***	w^{1118}; P{UAS-$TTR^{V30M-HA}$}	(Berg *et al.*, 2009)
**Balancers**	w^{1118};Sp/CyO;MKRS/TM6b, Tb	in-house
**GMR>*TTR***	GMR-Gal4/GMR-Gal4;+/+;UAS-TTR/UAS-TTR	This study (Fig. 1, S1)
**GMR>*TTR^{V30M}***	GMR-Gal4/GMR-Gal4;+/+;UAS-TTR^{V30M}/UAS-TTR^{V30M}	This study (Fig. 1-4, S1, S2)
**GMR>*GFP***	GMR-Gal4/GMR-Gal4;+/+;UAS-GFP/UAS-GFP	This study (Fig. 1, S1)
**GMR>GFP;*TTR^{V30M}***	GMR-Gal4/+;UAS-GFP/+; UAS-TTR^{V30M}/+	This study (Fig. S1)
**GMR>*Lifeact;TTR^{V30M}***	GMR-Gal4/+;UAS-Lifeact/+;UAS-TTR^{V30M}/+	This study (Fig. 2, 4, S2)
Cdc42^3	w^1 sn^3 $Cdc42^3$/FM6	Bloomington 7337
Pak^{11}	Pak^{11}/TM3, Sb^1	Bloomington 8810
Rac1$^{\Delta}$ Rac2$^{\Delta}$	y^1 w*; $Rac1^{J11}$ $Rac2^{\Delta}$ P{FRT(whs)}2A/TM6B, Tb+	Bloomington 6677
Rho1^{72F}	y^1 w*; $Rho1^{72F}$/CyO	Bloomington 7326
Rok^2	y^1 w^{1118} Rok^2 P{neoFRT}19A/FM7c	Bloomington 6666
Limk1^2	w* $LIMK1^2$	Bloomington 59033
tsr^{N121}	w*; P{FRT(w^{hs})}G13 tsr^{N121}/CyO	Bloomington 9109
chic^{221}	$chic^{221}$ cn^1/CyO; ry^{506}	Bloomington 4892
